# Memory Deficits in Children with Developmental Dyslexia: A Reading-Level and Chronological-Age Matched Design

**DOI:** 10.3390/brainsci11010040

**Published:** 2021-01-01

**Authors:** Giulia Lazzaro, Cristiana Varuzza, Floriana Costanzo, Elisa Fucà, Silvia Di Vara, Maria Elena De Matteis, Stefano Vicari, Deny Menghini

**Affiliations:** 1 Child and Adolescent Neuropsychiatry Unit, Department of Neuroscience, Bambino Gesù Children’s Hospital, IRCCS, 00165 Rome, Italy; giulia.lazzaro@opbg.net (G.L.); cristiana.varuzza@opbg.net (C.V.); floriana.costanzo@opbg.net (F.C.); elisa.fuca@opbg.net (E.F.); silvia.divara@opbg.net (S.D.V.); mariaelena.dematteis@opbg.net (M.E.D.M.); stefano.vicari@opbg.net (S.V.); 2 Department of Human Science, LUMSA University, 00193 Rome, Italy; 3 Department of Life Science and Public Health, Università Cattolica del Sacro Cuore, 00168 Rome, Italy

**Keywords:** multifactorial deficit, short-term memory, long-term memory, experience, causal deficit

## Abstract

Developmental Dyslexia (DD) is considered a multifactorial deficit. Among the neurocognitive impairments identified in DD, it has been found that memory plays a particularly important role in reading and learning. The present study aims to investigate whether short-term memory (STM) and long-term memory (LTM) deficits could be related to poor reading experience or could be causal factors in DD. To verify that memory deficits in DD did not simply reflect differences in reading experience, 16 children with DD were not only compared to 16 chronological age-matched children (CA) but also to 16 reading level-matched children (RL) in verbal, visual-object, and visual-spatial STM and LTM tasks. Children with DD performed as well as RL, but worse than CA in all STM tasks. Considering LTM, the three groups did not differ in Visual-Object and Visual-Spatial Learning tasks. In the Verbal LTM task, DD recalled significantly fewer words than CA but not RL, while CA and RL showed a similar performance. The present results suggest that when reading experience was equated, children with DD and typical readers did not differ in STM and LTM, especially in the verbal modality, weakening claims that memory has a causal effect in reading impairments.

## 1. Introduction

Reading is a highly interactive and integrative task that relies on visual-spatial capabilities to recognize and simultaneously elaborate letters within printed words [1] and on phonological skills to translate relevant orthographic units into their phonological representations [2]. The complexity of reading behaviour appears evident especially when a clinical condition such as developmental dyslexia (DD) is considered.

DD is a brain-based developmental disorder with ~7% of prevalence [3], characterized by severe and persistent impairments in the acquisition of reading despite appropriate instruction, adequate intelligence and intact sensory abilities [4]. Over the last few decades, a huge number of studies has revealed that DD may depend on multiple neurocognitive impairments, ranging from language-specific to cognitive-general deficits [5,6].

One of the most influential theories—the phonological core deficit hypothesis [7,8,9]—argues that DD may stem from deficits in phonological awareness. Many studies showed that lower phonological awareness predicts lower reading performance in typically reading children, and that phonological awareness deficits are often associated with impaired reading in DD [1]. There is also evidence for difficulties in low-level visual-temporal information processing, as the magnocellular deficit theory supports [10,11], as well as for visual-spatial attentional deficits [12,13] and visual-perceptual impairments [14,15,16].

Among these, the role of memory as a potential factor contributing to difficulties in the acquisition of reading has been largely investigated in the case of DD. Reading acquisition requires short-term memory (STM) in order to cross-modally and temporarily represent the order of a sequence of elements, such as letters of printed words [13]. Long-term memory (LTM), instead, plays a crucial role on the consolidation of sequence regularities in the phonological and orthographic input, leading to stable phonological and orthographic lexical representations and to a fast word recognition [17].

It is widely known that verbal STM and verbal working memory are typically reduced in children with DD compared to chronological age-matched children (CA) [18,19,20,21,22]. Beyond the verbal-related modality, children with DD often exhibit reduced visual-spatial/visual-perceptual STM and working memory compared to CA [18,23,24].

While not as extensive as STM, literature on LTM reveals deficits in individuals with DD [17,25]. Particularly, children with DD exhibit generalized deficits of LTM compared to CA regardless of age and in the verbal or non-verbal modalities. Moreover, these deficits predict reading difficulties [5].

The interpretation of memory impairments as one of the potential causes of DD is still challenging [26]. The major limitation of the aforementioned studies is the control group selection. Indeed, readers with DD were matched only to good readers of the same age but with a conceivably different level of reading experience. The poor reading experience that is inherent in being dyslexic could itself cause differences in neurocognitive processes. A reading-level matched design could be a way to overcome this main limitation—reading level-matched children (RL) allow to compare children with DD with younger children who have attained the same level of reading and, potentially, the same level of reading experience.

The current preliminary study aimed at disentangling whether reduced verbal and non-verbal STM and LTM, usually reported in DD, simply reflect differences in reading experience or constitute one of the core deficits of DD. Crucially for the aim of the present study, a CA and RL-matched design has been adopted and verbal, visual-object and visual-spatial STM and LTM tasks [27] have been administered to a group of children with DD, to RL, and CA. If children with DD perform worse than CA and equal to RL, lower reading skills could be linked to their reduced/(in)adequate reading experience and the result may open new questions on the relation between memory and reading deficits in DD.

## 2. Materials and Methods

### 2.1. Participants

Forty-eight right-handed Italian children were included in the current study, 16 with DD (Females, F/Males, M = 7/9), 16 RL (F/M = 9/7) and 16 CA (F/M = 5/11). The clinical diagnosis of DD was made based on the Diagnostic and Statistical Manual of Mental Disorders, 5th ed. criteria [4]. Children with DD showed reading speed or accuracy level at least 2 standard deviations (SD) below the mean of their chronological age. Children with DD were tested at the Child and Adolescent Psychiatry Unit of the Bambino Gesù Children’s Hospital (Italy), while participants of CA and RL groups were tested individually in a quiet room at their school.

All participants and their parents gave their informed consent in accordance with the Code of Ethics of the World Medical Association (Declaration of Helsinki), and the study was approved by the Bambino Gesù Children’s Hospital Ethical Committee (Protocol Number 486LB) on 5th October 2011.

Criteria for inclusion in the study were the following— (1) a normal or corrected-to-normal visual acuity; (2) non-verbal intelligence quotient (nvIQ) above 85 [28,29]; (3) no other significant comorbidity, such as attention deficit or hyperactivity disorder; (4) only for group with DD, not having undergone specific reading training.

Chronological age, nvIQ, and measures of reading (raw scores) were reported in Table 1 for each group. Children with DD did not differ for age from CA (F_2,45_ = 40.10, *p* = 0.63) and for reading abilities from RL (word reading errors (F_2,45_ = 13.10, *p* = 0.24), word reading times (F_2,45_ = 3.9, *p* = 0.24), and non-word reading times (F_2,45_ = 19.29, *p* = 0.47)). The three groups did not differ for nvIQ (F_2,45_ = 1.27, *p* = 0.29).

### 2.2. Measures

The neuropsychological tasks were administered to each participant individually in a pseudorandom way in 3 testing sessions carried out on separate days. In the first session, nvIQ and reading measures were assessed. The remaining tasks were administered in a random order. The battery concerned general intelligence as well as reading, verbal, visual-object and visual-spatial STM and LTM tasks. Every session took about one hour and a half.

#### 2.2.1. Reading Assessment

Reading abilities were measured by the word and non-word reading subtests from the clinical norm-referenced “Battery for the evaluation of Developmental Dyslexia and Dysorthography” [30]. In the first subtest, children had to read aloud 4 lists of 28 concrete and abstract, high-, or low-frequency words (4–8 letters in length). In the second task, participants had to read 4 lists of 16 legal non-words (5–9 letters in length). For both word and non-word subtests, the total errors were calculated adding the number of words or non-words read incorrectly of each list. As well, for both word and non-word subtests, the total times were computed by adding the time (in seconds) of each list, and therefore, considered as the total amount of seconds for each task.

#### 2.2.2. Memory Assessment

##### Verbal, Visual-Object, and Visual-Spatial STM

In order to assess STM, the Verbal, Visual-Object, and Visual-Spatial Span tasks from the norm-referenced “Battery for the evaluation of Memory” [27] were administered.

The Verbal Span Task consists of a list of eight, two-syllables low-frequency words. In the first block, the examiner read aloud two words at a rate of one item per second. The participants were required to repeat the two words in the same order. Then, four additional strings of two words were presented. If the child was successful in at least three of the five sequences, a sequence one word longer was presented. If the child failed (less than three correct answers in a block), the task was discontinued. The same procedure was used for sequences of increasing length (up to a maximum of seven words).

In the Visual-Object Span Task, the experimental material consisted of seven complex geometric figures depicted in high contrast colours. Two figures were presented, one at time, for 2 seconds at the centre of the computer screen. After the disappearance of the second figure, the two figures were presented aligned in the centre of the screen in a random position and the participant was asked to indicate the order in which they appeared. In this case as well, if the child was successful in at least three of the five trials, a sequence one figure longer was presented, and the task continued until a maximum of seven figures have been presented.

A similar procedure was used for the Visual-Spatial Span Task. In this case, the material consisted of a non-verbalizable geometric shape that appeared for 2 seconds in one of seven possible positions on the computer screen. Then, two empty cells were presented in the same spatial positions as before and the child had to indicate the order in which the stimuli appeared. If the child was successful in at least three of five two-position sequences, a sequence one block longer was presented. In this case too, the same testing procedure was used for sequences of increasing length (up to a maximum of seven spatial positions).

The score of all span tasks was computed by assigning 0.5 to each sequence of items correctly reproduced by the participant (maximum score = 17.5).

##### Verbal, Visual-Object, and Visual-Spatial LTM

In order to assess LTM, the Verbal, Visual-Object, and Visual-Spatial Learning tasks from the norm-referenced “Battery for the evaluation of Memory” [27] were administered.

In the Verbal Learning Task, participants were given a list of fifteen semantically unrelated words. The list was presented orally by the examiner for three consecutive times (Trials I, II, III) and each time the participant was asked to immediately repeat (1 min interval) as many items as possible in any order. The number of words recalled on average across the Trials (I, II, III) was scored and considered in the statistical analyses.

In the Visual-Object Learning Task, during the study phase, fifteen coloured drawings of common objects (e.g., a tree, a knife, a flower) were shown to the participants. Each figure was presented individually for 5 seconds. During the test phase, which immediately followed the study phase (1 min interval), fifteen pages were presented to the participant in succession. Four different versions of the same object (e.g., four trees, four knives, four flowers) were depicted on each page; only one of the four was the same as the target object in the study phase while the other three were physically different distracters. The participant was asked to indicate the object, which had been previously studied. The choice to use different representations of the same object as distracters in the recognition phase was aimed at minimizing the adoption of verbal coding and/or retrieval strategy. Study and test phases were presented for three consecutive times (Trials I, II, III). The number of average elements correctly recognized across the Trials (I, II, III) was scored and considered in the statistical analyses.

In the Visual-Spatial Learning Task, fifteen figures of common objects were also presented in the study phase. In this case, however, the pages were divided into four quadrants and each figure was positioned in one of the quadrants. During the test phase, which immediately follows the study phase (1 min interval), the target stimuli were presented individually, and the participant was asked to indicate the position occupied by the figure on an empty page sub-divided into four quadrants. Similar to the other two tasks, the test was repeated three times (Trials I, II, III). The number of average elements correctly recognized across the Trials (I, II, III) was scored and considered in the statistical analyses.

### 2.3. Statistical Analyses

To test whether the scores of STM tasks differed between groups, a Multivariate Analysis of Variance (MANOVA) with Group (DD, RL, CA) as between-subject factors and Type of Span Task (Verbal vs. Visual-Object vs. Visual-Spatial) as within-subject factors was run.

To verify whether the scores of LTM tasks differed between groups, a MANOVA with Group (DD, RL, CA) as between-subject factors and Type of Learning Task (Verbal vs. Visual-Object vs. Visual-Spatial) as within-subject factors was run.

The potential effects of age were considered by including the participants’ age as a covariate. Post hoc analyses were performed using Tukey HSD test. Partial eta squares (ηp^2^) were reported as measures of effect size.

For all analyses, a *p* value ≤ 0.05 was considered statistically significant.

To investigate the relation between reading and memory measures, partial correlations with Inefficiency Index of Word or Non-word reading as independent variables, scores of STM and LTM as dependent variables and age as a control variable were run. The two separate Inefficiency Reading Indexes were calculated for word and non-word as the ratio between word or non-word reading speed (in seconds) and accuracy rate (number of words or non-words correctly read by the total number of words or non-words of the list). A comprehensive Inefficiency Reading Index, with combined measure of speed and accuracy, was considered to reduce the number of comparisons between reading and memory variables.

After correcting statistical significance for multiple comparisons ((2 Inefficiency Indexes of Word and Non-word and 6 memory measures (3 STM measures: Verbal, Visual-Object, Visual-Spatial Span Tasks; 3 LTM measures: Verbal, Visual-Object, Visual-Spatial Learning Tasks)), a *p* value ≤ 0.004 was considered (after Bonferroni’s Correction, 0.05/12 = 0.004).

## 3. Results

### 3.1. Verbal, Visual-Object, and Visual-Spatial STM

After controlling for age, results showed a significant main effect of Group (F_2,44_ = 11.26, *p* = 0.0001, η*p*^2^ = 0.34). Post-hoc comparisons documented overall higher scores of CA (M ± SD: 8.82 ± 1.39) compared to DD (7.07 ± 1.48, *p* < 0.001) and to RL (7.63 ± 1.63, *p* < 0.01). No significant differences were found between DD and RL (*p* = 0.29). The main effect of Type of Span Task (F_2,88_ = 0.17, *p* = 0.84, η*p*^2^ = 0.003) and the interaction Group × Type of Span Task (F_4,88_ = 0.14, *p* = 0.97, η*p*^2^ = 0.01) was not significant.

### 3.2. Verbal, Visual-Object, and Visual-Spatial LTM

After controlling for age, results showed that the main effect of Type of Learning Task (F_2,88_ = 1.15, *p* = 0.32, η*p*^2^ = 0.03) was not significant, while the main effect of Group (F_2,44_ = 5.41, *p* = 0.008, η*p*^2^ = 0.20) was significant. Post-hoc comparisons revealed that CA (11.39 ± 1.55) recalled on the average more items than children with DD (10.35 ± 2.09, *p* < 0.001) and RL (10.29 ± 1.84, *p* < 0.001) and that DD and RL did not differ in the number of recalled items (*p* = 0.98).

The Group × Type of Learning Task interaction (F_4,88_ = 2.51, *p* = 0.048, η*p*^2^ = 0.10) was found to be significant, after controlling for age. As Table 2 shows, post-hoc comparisons in Verbal Learning Task documented that DD recalled on average less items than CA (*p* = 0.003) but not than RL (*p* = 0.45) and that CA and RL did not differ in the number of recalled items (*p* = 0.72). In Visual-Object and Visual-Spatial Learning Tasks, no differences emerged between the three groups (*p* always >0.10).

### 3.3. Correlations between Reading and Memory

As shown in Table 3, after controlling for age, Word Inefficiency Index was significantly and negatively correlated to Verbal STM (*p* < 0.0001), meaning that worse word reading was associated with lower scores in verbal STM. Similarly, Word (*p* < 0.0001) and Non-word (*p* = 0.003) Inefficiency Indexes were significantly and negatively correlated to Verbal LTM, meaning that worse word and non-word reading was associated with lower scores in verbal LTM.

No further significant correlations were found, after considering statistical significance correction for multiple comparisons (*p* > 0.004).

## 4. Discussion

The current study aims at investigating whether impairments of STM and LTM usually reported in children with DD could be related to reduced or (in)adequate reading experience or constitute one of the core deficits of DD. To this aim, the effects of reading experience on different LTM and STM domains were investigated adopting a RL and CA-matched groups design.

Within STM, after controlling for age, children with DD obtained significantly lower scores than CA not only on a verbal span task but also on tasks assessing visual-object and visual-spatial span. Crucially for the aim of the present study, controlling for age, children with DD did not differ from the RL in any aspect of STM assessed.

The present results could be interpreted under a neurocognitive perspective, according to which memory deficits in children with DD are partly shaped by poor reading experience. Converging opinion articles [26,31,32] supported the idea that neurocognitive impairments, usually reported in individuals with DD, could be considered as secondary consequences of reduced or (in)adequate reading experience. In line with the so-called “Matthew effect” [33], according to which the ‘rich get richer’ during the development of reading, children who read more develop better phonological awareness, which in turn enhances phonological STM and working memory and, reading itself. However, children with DD accumulate far less experience of reading, and of the related processes involved, such as phonological processing, visual analysis, visual-spatial attention, STM, and working memory. Therefore, after years of poor exposure to reading, children with DD would show atypical development of several skills. In line with this hypothesis, a pioneering longitudinal study [34] showed that the acquisition of reading promoted growth of phonological processing and verbal STM, and that phonological skills in turn lead to the development of visual STM. A more recent longitudinal study [35] showed that reading proficiency at 6 years predicts verbal STM abilities, as measured by non-word repetition task, even after controlling for language skills.

A way to better understand the role of experience on the development of neurocognitive processes involved in reading, such as STM, is to compare adults who could not attend school during childhood and who either remained illiterate [31,36,37,38,39] or acquired literacy during adulthood (ex-illiterates) [40,41]. Findings revealed that illiterates generally performed more poorly than literates on memory tasks, such as non-word repetition [42,43], verbal paired associates [44,45], digits backwards [44] and complex figure drawing [46]. Although still few in number, findings on children [47] confirmed that, after controlling for CA, socio-economic status and associated learning disabilities, illiterates performed worse than literate in several tasks, including verbal and visual memory coding, and delayed recall. Therefore, comparisons between illiterates and literates could be partially interpreted in terms of differences in reading experience, as illiterates have never been taught to read, thus part of neurocognitive processes strictly related to reading has not been developed as well. Intriguingly, comparisons between illiterates and ex-illiterates on STM could further substantiate the hypothesis that aspects of memory development might be at least partially influenced by literacy. Findings on adults [40,41] documented that illiterates displayed lower performance on immediate serial recall of pictures, forward digit-span and listening span compared to ex-illiterates.

Overall, learning to read seems to partially reinforce certain abilities, such as verbal and visual memory. However, we are aware that improvement in memory across life-span could be linked with education more broadly rather than with literacy only. Indeed, it is possible that education in general leads to enhanced memory abilities rather than literacy specifically.

Although poor reading experience is a plausible explanation for reduced STM abilities in our children with DD as compared with CA but not to RL, we cannot ignore that memory deficits could reflect a general maturational lag. It is widely documented that memory undergoes important changes across childhood and adolescence [48,49], along with a number of structural maturational processes in the brain [50,51]. Functional neuroimaging findings [52] reported that working memory capacity was correlated with brain activity in the fronto-parietal network and that older children showed higher activation than the younger children did. These age-related differences on STM capacity were evident in our groups when performances of CA and RL were compared. Indeed, CA showed higher memory capacity compared to RL and to children with DD, regardless of the type of the task considered. We cannot exclude that differences on STM tasks between CA and children with DD could be the result of a developmental delay of STM and of related brain regions in children with DD, which in turn performed as well as younger typical readers (i.e., RL).

In summary, the fact that our group of children with DD exhibited a general impairment in STM compared to CA but not to RL, regardless of the type of the task considered, could be interpreted as a result of limited reading exposure or inadequate cognitive maturation correlated with brain-based development. Our data were in line with previous studies showing verbal STM deficits in individuals with DD in CA-matched design studies—see the review by Steve Majerus and Nelson Cowan [21]. Of importance, as our children with DD performed as well as RL, the hypothesis that verbal STM deficits are an underlying causal factor of DD may still be under debate. Further, the present study confirmed prior findings in which children with DD were matched to CA, showing that STM deficits were not limited to verbal aspects but extended also to visual-spatial and visual-perceptual stimuli [1,18].

Within LTM, after controlling for age, the two control groups (CA and RL) did not show any significant difference in the verbal task, while children with DD recalled significantly less words than CA and comparable number of words to that of RL. However, in visual-object and visual-spatial tasks the three groups did not differ. Differently from what emerged from STM, LTM abilities did not seem to be linked to memory development or to reading experience because the two groups of controls (CA and RL) did not differ in Verbal, Visual-Object nor Visual-Spatial Learning Tasks despite different ages and levels of education. The fact that children with DD recalled a similar number of words of RL did not support the hypothesis that verbal LTM impairment represents one of the multifactorial deficits which plays a role in DD. Indeed, it would have been hypothesized that a core deficit in verbal LTM occurred in DD only if children with DD had performed worse than younger typical readers (i.e., RL). Since children with DD did not perform worse than RL after controlling for age, the role for LTM in DD is still causally ambiguous.

In line with previous literature [17,21], analyses of correlations between reading and memory measures showed that poor word and non-word reading are related to reduced STM and LTM memory abilities within the verbal domains. Namely, controlling for age, children with lower verbal span show worse word reading and children with lower verbal LTM show worse word and non-word reading. Our findings sustain the interpretation that verbal memory and reading experience are closely related, after controlling for age.

Our study has some limitations. The number of participants is limited and collecting data from larger groups is needed. Another limitation is that verbal, visual-object and visual-spatial performances in our children are not controlled for language and visual-spatial abilities. The present study also lacks parental socio-economic assessment which might have influenced the level of reading experience among the three groups. In order to better understand the relation between reading, STM and LTM in DD, longitudinal studies following children over the learning trajectory are also urgently needed to establish temporal cause versus effect. Longitudinal design should include the key neurocognitive processes involved in reading to verify the single contribution and their mutual relations in influencing atypical reading development. Finally, training studies are required to test of causation in reading of neurocognitive deficits. If an atypical process caused reading deficits in children with DD, then providing training in such a process should ameliorate reading.

## 5. Conclusions

In summary, our results showed that when reading experience is controlled, children with DD and typical readers did not differ in memory and the causal effect of memory in reading impairment has not been ascertained so far.

Although preliminary, the present study provides new insight into our understanding of memory abilities in children with DD and the relationship between reading experience and neurocognitive processes involved in reading.

## Figures and Tables

**Table 1:** Participant characteristics by group.

	DD (*n* = 16)	RL (*n* = 16)	CA (*n* = 16)	Post-Hoc Comparisons
Age ^a^	12.78 (1.32)	9.92 (1.45)	13.14 (0.82)	DD > RL *** DD = CA RL < CA ***
nvIQ ^b^	107.5 (8.56)	103.75 (10.25)	109.25 (10.97)	DD = RL = CA
Word reading errors ^c^	3.17 (1.76)	2.38 (1.46)	0.68 (0.87)	DD = RL DD > CA *** RL > CA **
Word reading times ^d^	112.44 (33.19)	98.25 (25.32)	55.19 (6.36)	DD = RL DD > CA *** RL > CA ***
Non-word reading errors ^c^	8.81 (5.10)	4.56 (3.37)	2.44 (1.41)	DD > RL ** DD > CA *** RL = CA
Non-word reading times ^d^	88.94 (33.52)	79.25 (20.52)	41.0 (7.31)	DD = RL DD > CA *** RL > CA ***

** *p* < 0.01; *** *p* < 0.001; ^a^ in years; ^b^ non-verbal intelligence quotient; ^c^ total number of word or non-word read incorrectly; ^d^ total time in seconds. DD = children with Developmental Dyslexia; RL = Reading Level-matched children; CA = Chronological Age-matched children.

**Table 2:** Mean and SD for Verbal, Visual-Object and Visual-Spatial Learning Tasks of LTM by groups.

	Groups			
Learning Tasks	DD Mean (SD)	RL Mean (SD)	CA Mean (SD)	Post-Hoc Comparisons
Verbal	7.79 (2.15)	8.96 (1.54)	9.92 (1.81)	DD < CA ** DD = RL CA = RL
Visual-Object	11.19 (1.97)	10.25 (1.54)	11.19 (1.45)	DD = RL = CA
Visual-Spatial	12.06 (2.13)	11.65 (2.43)	13.06 (1.37)	DD = RL = CA

** *p* < 0.01. DD = children with Developmental Dyslexia; RL = Reading Level-matched children; CA = Chronological Age-matched children.

**Table 3:** Correlations (Pearson’s *r*) between the Inefficiency Index of Word and Non-word and scores of STM and LTM tasks, controlling for age.

	Inefficiency Index	
	Word	Non-word
	*r*	*r*
**STM**		
**Verbal Span Tasks**	−0.53 ***	−0.40 **
**Visual-Object Span Tasks**	−0.33 *	−0.14
**Visual-Spatial Span Tasks**	−0.21	−0.11
**LTM**		
**Verbal Learning Tasks**	−0.52 ***	−0.42 ***
**Visual-Object Learning Tasks**	0.09	0.05
**Visual-Spatial Learning Tasks**	−0.11	−0.01

* *p* < 0.05, ** *p* < 0.01, *** survived after Bonferroni’s Correction, *p* ≤ 0.004. STM = Short-Term Memory; LTM = Long-Term Memory.

## Data Availability

The data that support the findings of this study are available upon reasonable request.

## References

[1] Valdois S., Reilhac C., Ginestet E., Bosse M.L. (2020). Varieties of Cognitive Profiles in Poor Readers: Evidence for a VAS-Impaired Subtype. J. Learn. Disabil..

[2] Richlan F. (2019). The Functional Neuroanatomy of Letter-Speech Sound Integration and Its Relation to Brain Abnormalities in Developmental Dyslexia. Front. Hum. Neurosci..

[3] Peterson R.L., Pennington B.F. (2015). Developmental Dyslexia. Annu. Rev. Clin. Psychol..

[4] American Psychiatric Association (2013). Diagnostic and Statistical Manual of Mental Disorders.

[5] Menghini D., Finzi A., Benassi M., Bolzani R., Facoetti A., Giovagnoli S., Ruffino M., Vicari S. (2010). Different underlying neurocognitive deficits in developmental dyslexia: A comparative study. Neuropsychologia.

[6] D’Mello A.M., Gabrieli J.D.E. (2018). Cognitive Neuroscience of Dyslexia. Lang. Speech Hear. Serv. Sch..

[7] Snowling M. (2000). Dyslexia.

[8] Vellutino F.R., Fletcher J.M., Snowling M.J., Scanlon D.M. (2004). Specific reading disability (dyslexia): What have we learned in the past four decades?. J. Child Psychol. Psychiatry.

[9] Melby-Lervåg M., Lyster S.-A.H., Hulme C. (2012). Phonological skills and their role in learning to read: A meta-analytic review. Psychol. Bull..

[10] Schulte-Körne G., Bruder J. (2010). Clinical neurophysiology of visual and auditory processing in dyslexia: A review. Clin. Neurophysiol..

[11] Vidyasagar T.R., Pammer K. (2010). Dyslexia: A deficit in visuo-spatial attention, not in phonological processing. Trends Cogn. Sci..

[12] Franceschini S., Gori S., Ruffino M., Pedrolli K., Facoetti A. (2012). A Causal Link between Visual Spatial Attention and Reading Acquisition. Curr. Biol..

[13] Bosse M.-L., Tainturier M.J., Valdois S. (2007). Developmental dyslexia: The visual attention span deficit hypothesis. Cognition.

[14] Stenneken P., Egetemeir J., Schulte-Körne G., Müller H.J., Schneider W.X., Finke K., Bogon J. (2011). Slow perceptual processing at the core of developmental dyslexia: A parameter-based assessment of visual attention. Neuropsychologia.

[15] Giovagnoli G., Vicari S., Tomassetti S., Menghini D. (2016). The Role of Visual-Spatial Abilities in Dyslexia: Age Differences in Children’s Reading?. Front. Psychol..

[16] Wang Z., Cheng-Lai A., Song Y., Cutting L., Jiang Y., Lin O., Meng X., Zhou X. (2014). A Perceptual Learning Deficit in Chinese Developmental Dyslexia as Revealed by Visual Texture Discrimination Training. Dyslexia.

[17] Bogaerts L., Szmalec A., Hachmann W.M., Page M.P., Duyck W. (2015). Linking memory and language: Evidence for a serial-order learning impairment in dyslexia. Res. Dev. Disabil..

[18] Menghini D., Finzi A., Carlesimo G.A., Vicari S. (2011). Working Memory Impairment in Children With Developmental Dyslexia: Is it Just a Phonological Deficity?. Dev. Neuropsychol..

[19] Wang S., Gathercole S.E. (2013). Working memory deficits in children with reading difficulties: Memory span and dual task coordination. J. Exp. Child Psychol..

[20] Zhao J., Yang Y., Song Y.-W., Bi H.-Y. (2015). Verbal Short-Term Memory Deficits in Chinese Children with Dyslexia may not be a Problem with the Activation of Phonological Representations. Dyslexia.

[21] Majerus S., Cowan N. (2016). The Nature of Verbal Short-Term Impairment in Dyslexia: The Importance of Serial Order. Front. Psychol..

[22] Cowan N., Hogan T.P., Alt M., Green S., Cabbage K.L., Brinkley S., Gray S. (2017). Short-term Memory in Childhood Dyslexia: Deficient Serial Order in Multiple Modalities. Dyslexia.

[23] Varvara P., Varuzza C., Sorrentino A.C.P., Vicari S., Menghini D. (2014). Executive functions in developmental dyslexia. Front. Hum. Neurosci..

[24] Valdois S., Lassus-Sangosse D., Lobier M. (2012). Impaired Letter-String Processing in Developmental Dyslexia: What Visual-to-Phonology Code Mapping Disorder?. Dyslexia.

[25] Bogaerts L., Szmalec A., De Maeyer M., Page M.P., Duyck W. (2016). The involvement of long-term serial-order memory in reading development: A longitudinal study. J. Exp. Child Psychol..

[26] Goswami U. (2015). Sensory theories of developmental dyslexia: Three challenges for research. Nat. Rev. Neurosci..

[27] Vicari S. (2007). PROMEA, Prove di Memoria e Apprendimento per l’età evolutive.

[28] Raven J.C. (2008). CPM, Coloured Progressive Matrices, series A, AB, B.

[29] Raven J.C. (2009). SPM, Coloured Progressive Matrices, series A, AB, B.

[30] Sartori G., Job R., Tressoldi P.E. (2007). DDE-2 Batteria Per la Valutazione Della Dislessia e Della Disortografia Evolutiva-2.

[31] Demoulin C., Kolinsky R. (2015). Does learning to read shape verbal working memory?. Psychon. Bull. Rev..

[32] Huettig F., Lachmann T., Reis A., Petersson K.M. (2018). Distinguishing cause from effect—Many deficits associated with developmental dyslexia may be a consequence of reduced and suboptimal reading experience. Lang. Cogn. Neurosci..

[33] Stanovich K.E. (1986). Matthew Effects in Reading: Some Consequences of Individual Differences in the Acquisition of Literacy. Read. Res. Q..

[34] Ellis N. (1990). Reading, phonological skills and short-term memory: Interactive tributaries of development. J. Res. Read..

[35] Nation K., Hulme C. (2010). Learning to read changes children’s phonological skills: Evidence from a latent variable longitudinal study of reading and nonword repetition. Dev. Sci..

[36] Ardila A., Bertolucci P.H., Braga L.W., Castro-Caldas A., Judd T., Kosmidis M.H., Matute E., Nitrini R., Ostrosky-Solis F., Rosselli M. (2010). Illiteracy: The Neuropsychology of Cognition Without Reading. Arch. Clin. Neuropsychol..

[37] Silva C., Faísca L., Ingvar M., Petersson K.M., Reis A. (2012). Literacy: Exploring working memory systems. J. Clin. Exp. Neuropsychol..

[38] Smalle E.H.M., Szmalec A., Bogaerts L., Page M.P., Narang V., Misra D., Araújo S., Lohagun N., Khan O., Singh A. (2019). Literacy improves short-term serial recall of spoken verbal but not visuospatial items—Evidence from illiterate and literate adults. Cognition.

[39] Horowitz-Kraus T., Hutton J.S. (2015). From emergent literacy to reading: How learning to read changes a child’s brain. Acta Paediatr..

[40] Morais J., Bertelson P., Cary L., Alegria J. (1986). Literacy training and speech segmentation. Cognition.

[41] Kosmidis M.H., Zafiri M., Politimou N. (2011). Literacy versus formal schooling: Influence on working memory. Arch. Clin. Neuro-psychol..

[42] Kosmidis M.H., Tsapkini K., Folia V. (2006). Lexical Processing in Illiteracy: Effect of Literacy or Education?. Cortex.

[43] Petersson K.-M., Reis A., Ingvar M. (2001). Cognitive processing in literate and illiterate subjects: A review of some recent behavioral and functional neuroimaging data. Scand. J. Psychol..

[44] Montiel T., Matute E. (2006). La relación entre alfabetización y la escolarización con el desempeno en tareas verbales. Lectura y Diversidad Cultural.

[45] Reis A., Castro-Caldas A. (1997). Illiteracy: A bias for cognitive development. J. Int. Neuropsychol. Soc..

[46] Ardila A., Rosselli M., Rosas P. (1989). Neuropsychological assessment in illiterates: Visuospatial and memory abilities. Brain Cogn..

[47] Matute E., Montiel T., Pinto N., Rosselli M., Ardila A., Zarabozo D. (2012). Comparing cognitive performance in illiterate and literate children. Int. Rev. Educ..

[48] Gathercole S.E., Pickering S.J., Ambridge B., Wearing H. (2004). sThe Structure of Working Memory From 4 to 15 Years of Age. Dev. Psychol..

[49] Klingberg T. (2006). Development of a superior frontal–intraparietal network for visuo-spatial working memory. Neuropsychologia.

[50] Giedd J.N., Blumenthal J., Jeffries N.O., Castellanos F.X., Liu H., Zijdenbos A., Paus T., Evans A.C., Rapoport J.L. (1999). Brain development during childhood and adolescence: A longitudinal MRI study. Nat. Neurosci..

[51] Lebel C., Beaulieu C. (2011). Longitudinal Development of Human Brain Wiring Continues from Childhood into Adulthood. J. Neurosci..

[52] Klingberg T., Forssberg H., Westerberg H. (2002). Training of Working Memory in Children With ADHD. J. Clin. Exp. Neuropsychol..

